# Advanced Molecular Surveillance of Hepatitis C Virus

**DOI:** 10.3390/v7031153

**Published:** 2015-03-13

**Authors:** Livia Maria Gonçalves Rossi, Alejandro Escobar-Gutierrez, Paula Rahal

**Affiliations:** 1 Department of Biology, Institute of Bioscience, Language and Exact Science, Sao Paulo State University, Sao Jose do Rio Preto, SP 15054-000, Brazil; E-Mail: rahalp@yahoo.com.br; 2 Instituto de Diagnostico y Referencia Epidemiologicos, Mexico City 01480, Mexico; E-Mail: aescobargutierrez@yahoo.com

**Keywords:** Hepatitis C virus, advanced molecular surveillance, molecular epidemiology, molecular characterization, host factors

## Abstract

Hepatitis C virus (HCV) infection is an important public health problem worldwide. HCV exploits complex molecular mechanisms, which result in a high degree of intrahost genetic heterogeneity. This high degree of variability represents a challenge for the accurate establishment of genetic relatedness between cases and complicates the identification of sources of infection. Tracking HCV infections is crucial for the elucidation of routes of transmission in a variety of settings. Therefore, implementation of HCV advanced molecular surveillance (AMS) is essential for disease control. Accounting for virulence is also important for HCV AMS and both viral and host factors contribute to the disease outcome. Therefore, HCV AMS requires the incorporation of host factors as an integral component of the algorithms used to monitor disease occurrence. Importantly, implementation of comprehensive global databases and data mining are also needed for the proper study of the mechanisms responsible for HCV transmission. Here, we review molecular aspects associated with HCV transmission, as well as the most recent technological advances used for virus and host characterization. Additionally, the cornerstone discoveries that have defined the pathway for viral characterization are presented and the importance of implementing advanced HCV molecular surveillance is highlighted.

## 1. Introduction

Hepatitis C virus (HCV) infection is an important global public health problem. Approximately 180 million people are currently infected with HCV [1], and an alarming number of new infections occur annually [2,3]. The prevalence of hepatitis C varies significantly worldwide, imposing an important burden in highly endemic countries [2]. HCV infection is commonly associated with chronic liver disease, which frequently results in the advanced stages of cirrhosis and hepatocellular carcinoma (HCC) following years of silent infection [4,5].

**Figure 1:**
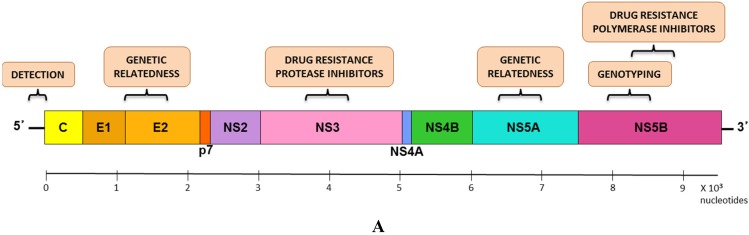

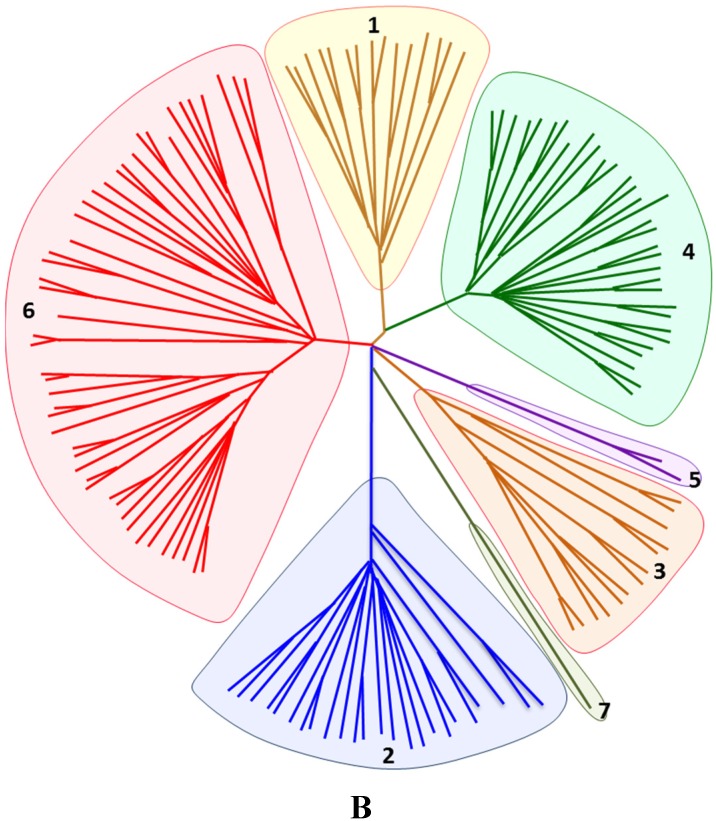
HCV genome organization and genotypes. (**A**) HCV genome organization is shown. The most common regions used for molecular analyses and genetic relatedness studies are highlighted; (**B**) HCV representative strains belonging to all seven genotypes and all different subtypes are depicted. Sequences from the NS5B region were chosen to illustrate the maximum diversity within a subtype. The neighbor joining tree was constructed using maximum composite likelihood nucleotide distances using MEGA5 and 1000 replications for bootstrapping.

HCV is a single-stranded, positive-sense, enveloped flavivirus. The viral RNA genome is ~9.6 kb in length and contains a single open reading frame encoding a large polyprotein. The polyprotein is processed by viral and host proteases, resulting in three structural proteins and seven nonstructural proteins (Figure 1A) [6,7].

HCV molecular evolution plays an important role in virus transmission, dictating in many ways the outcome of disease and therapy. Based on the nucleotide variability in the viral genome, seven HCV genotypes and multiple subtypes have been recognized (Figure 1B) [8]. These genotypes exhibit a characteristic distribution [9]: genotypes 1–3 are distributed worldwide, while genotypes 4 and 5 are mainly found in Africa and genotype 6 is endemic in Asia [9,10,11,12,13,14,15]. The high mutation rate is characteristic of HCV replication, resulting in a high degree of intrahost genetic diversity [16,17,18]. The molecular plasticity of HCV allows rapid rearrangement of the intrahost viral population under different selection pressures [19,20]. This remarkable genetic variability is one of the main factors that have prevented the development of a successful vaccine.

Molecular surveillance of hepatitis C is of the utmost importance for identification of HCV transmission networks. Molecular surveillance is a critical component of HCV outbreak investigations because tracking of infections is necessary for the identification of sources of infection and accurate identification of cases associated with transmission networks [21]. Additionally, molecular surveillance can provide important information about the capacity of HCV lineages to cause disease [21].

In addition to imposing important challenges for vaccine development, HCV genetic variability also represents a major issue for the establishment of genetic relatedness between cases associated with a common source of infection. The rapidly evolving nature of the virus results in significant changes in genetic makeup of the virus, which can prevent our ability to link cases by genetic similarity [16]. However, a high degree of genetic variability is also required to track infections and facilitates genetic relatedness studies which otherwise would be hampered [22]. Here, we present a historical review of the keystone discoveries that have propelled our understanding of the mechanisms controlling HCV transmission. Additionally, we discuss the most recent information on molecular aspects related to viral spread and novel approaches designed to assess HCV intrahost genetic variability for the establishment of genetic relatedness between cases. Finally, the implementation of advanced HCV molecular surveillance as an integral component of hepatitis C global epidemiology is highlighted.

## 2. Historical Timeline of HCV Characterization

**Figure 2:**
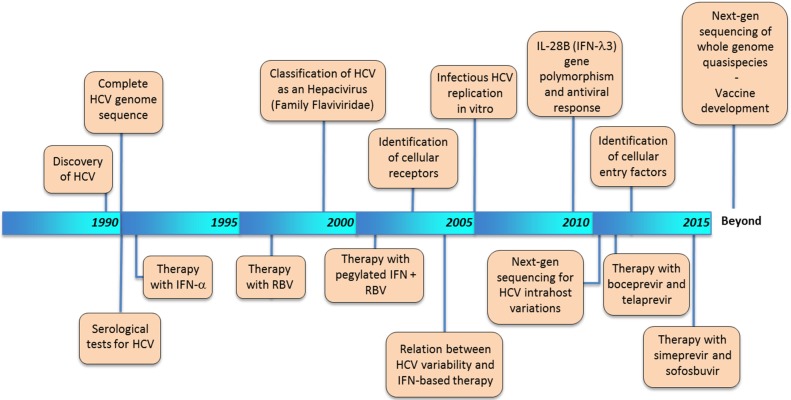
Chronological cornerstone developments in the field of HCV. The most relevant discoveries related to the study of HCV are shown in chronological order.

Since its discovery 25 years ago, the study of HCV has seen a number of cornerstone developments that have improved our understanding of HCV-related disease pathogenesis (Figure 2). The field of viral hepatitis initially began in the late 1950s with the coining of the term “infectious” or “serum” hepatitis [23]. This event was followed by the discovery of the hepatitis A (HAV) [24] and hepatitis B viruses (HBV) [25] in the late 1960s and early 1970s. However, our understanding of HCV infection dates to the late 1980s when researchers from the Centers for Disease Control and Chiron Corporation, led by Daniel Bradley and Michael Houghton, respectively, identified the virus in samples from experimentally infected chimpanzees [26]. Prior to its identification, the advent of serologic testing for HAV and HBV in the mid-1970s led to the realization that most infectious hepatitis cases were non-A, non-B (NANBH). These findings prompted the use of the chimpanzee as an animal model for the passage of the unidentified agent responsible for NANBH [27]. The subsequent development of serologic tests for the detection of HCV infection in the early 1990s allowed screening of the blood supply and successfully prevented transmission via transfusion [28]. HCV molecular divergence was recognized shortly after the discovery of the virus when Japanese strains were shown to be genetically distant from American strains [29]. Subsequently, the complete sequencing of the HCV genome allowed the elucidation of its organization [30,31,32]. Molecular characterization of the viral enzymes during the early 1990s served as the foundation for the development of successful anti-HCV therapies [18,33,34,35,36,37] and resulted in the constant reshaping of anti-HCV therapy based on interferon (IFN). The initial approval of alpha IFN (IFNα) for the treatment of HCV in 1991 was followed by the licensing of consensus IFN in 1997 and ribavirin (RBV) in 1998. In 2001, the introduction of pegylated IFN, a compound with significantly prolonged half-life in blood, increased the likelihood of a sustained virological response (SVR). Full recognition of the degree of genetic heterogeneity among HCV isolates was achieved during the mid-1990s [38,39], resulting in the proposed classification of *Hepaciviruses* as an independent genus within the *Flaviviridae* family representing a distant relative of the *Flavivirus* and *Pestivirus* genera [40]. Importantly, the fact that HCV diversity played an important role in interferon (IFN)-based therapy highlighted the relevance of assessing the genetic variability of the viral population *in vivo* [41,42].

An important advance for drug development was the generation of human hepatoma cell lines capable of replicating HCV [43,44,45,46]. Over the years, other important discoveries have been reported by several groups, especially relating to the characterization of viral proteins and their role in HCV replication [34,47,48,49]. Additionally, the characterization of several cellular receptors [50,51,52,53], and entry factors [54,55] has been reported.

The initial release of the first next generation sequencing (NGS) commercial platform in 2005 completely revolutionized the field of genetics. However, initial studies reporting the use of NGS approaches to assess HCV intrahost genetic variation emerged in late 2011 [56,57]. Since this time, a wealth of information has been generated using NGS to address a multitude of issues related to HCV transmission [58,59,60,61,62,63,64,65,66,67,68,69,70,71]. The challenge now resides in the study of whole genome (WG) genetic variation, which still represents a daunting task for the study of HCV molecular evolution since reconstruction of original haplotypes from NGS data is difficult [72,73,74].

In 2009, Ge *et al*. reported a strong association between polymorphisms in the promoter region of the interleukin-28B gene (also known as IFN-λ3) and the antiviral response based on IFN [75]. Subsequently, work conducted by other groups further confirmed the relationship between the IL-28B genotype and sustained viral response (SVR) [76,77]. The relevance of these reports cannot be understated, because they resulted in extensive work on human genetics and became an important component in the study of the pathogenesis of HCV-related disease. 

The arrival of the second generation of direct-acting antivirals (DAA) in 2011 resulted in improved SVR and a revolution in the field of anti-HCV therapy. Despite the development of drug resistance, the initial NS3-4A protease inhibitors (PI) telaprevir and boceprevir undoubtedly possessed advantages over dual IFN/RBV treatment [78]. The success of both drugs was subsequently eclipsed by the licensing of simeprevir and sofosbuvir [79,80,81,82]. The field of HCV therapy is rapidly evolving, and as a result, a large number of new antiviral drugs are currently being evaluated in advanced clinical trials [83].

The prevention of HCV infection has been hampered by the slow development of promising vaccine candidates. Phase I of two different vaccine trials have been completed. Testing of a prime-boost regimen with the candidate developed by GlaxoSmithKline after preliminary studies in the chimpanzee model has also been performed [84]. Importantly, conserved epitopes in HCV genotype 1 and 3 were successfully identified, suggesting the potential for cross-genotypic protection. Priming with this vaccine followed by boosting with a modified vaccinia virus is currently being evaluated in a phase I/II trial [85], with the goal of preventing persistence in HCV-naïve IDUs at high risk for infection.

## 3. Molecular Aspects of HCV Transmission

HCV transmission is a dynamic process that primarily occurs via parenteral routes and especially by unsafe injections, which have significantly facilitated virus spread [86]. The time of initial spread of HCV into Western countries and the population dynamics of the epidemic can only be indirectly inferred [13]. Current evidence supports a recent spread; however, the lack of samples prior to the Second World War has hindered our ability to reconstruct the HCV epidemic. Because vertical and sexual transmission contribute little to HCV spread, the restriction of HCV transmission through primarily parenteral routes implicates unsafe injections (*i.e.*, inadequate medical treatment, large-scale vaccination programs, blood transfusion, and injecting drug use) as the main vehicle for HCV transmission [13]. Limited information is available about the transmission of HCV prior to the invention of injections. However, cultural traditions, such as tattooing and scarification, were likely to have played an important role in the spread of HCV.

**Figure 3:**
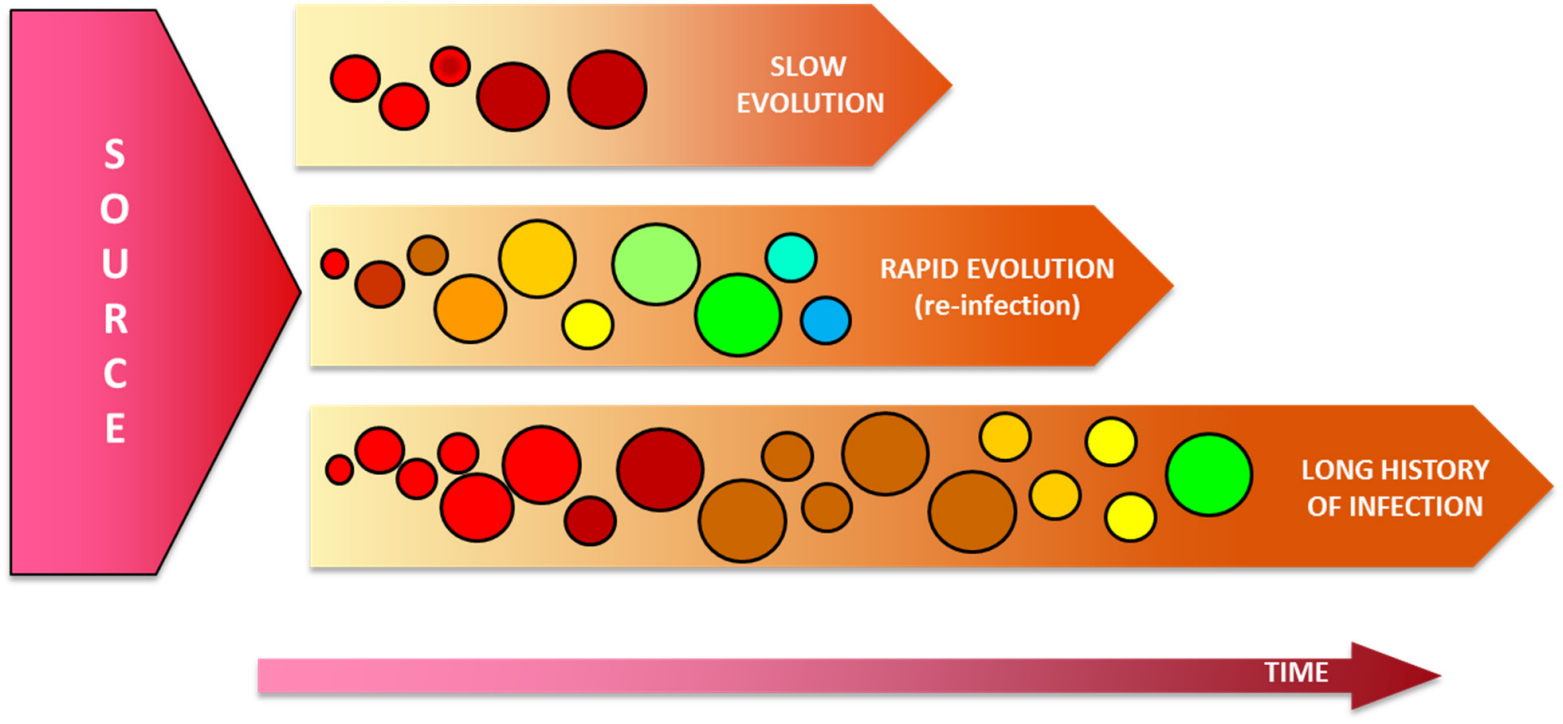
Rapid and slow HCV intrahost molecular evolution. The speed with which HCV evolves in different settings is shown. Viral populations are represented by circles. Viral populations are color coded to represent genetic diversity. The size of circles represents fluctuations in the size of viral populations over time. Generation of viral diversity during the course of infection is illustrated by the change in color of the viral population throughout the time.

HCV intrahost populations frequently exist as an ensemble of genetically distinct but closely related variants [18,87]. Analysis of HCV intrahost genetic variation is the basis of genetic relatedness and epidemiological studies as well as the identification of drug resistant mutations [88,89]. While in some instances genetic relatedness might be demonstrated with consensus sequencing of some viral subgenomic regions, HCV outbreak investigation commonly requires a much deeper analysis of the infecting viral population [16,90,91,92,93]. The rapidness with which HCV intrahost populations diverge significantly affects genetic relatedness studies because molecular epidemiological links can be lost between related cases in a relatively short period of time (Figure 3) [16,94,95,96]. Therefore, the use of more and longer subgenomic regions, such as the NS3, NS5A, and NS5B might help alleviate this issue.

**Figure 4:**
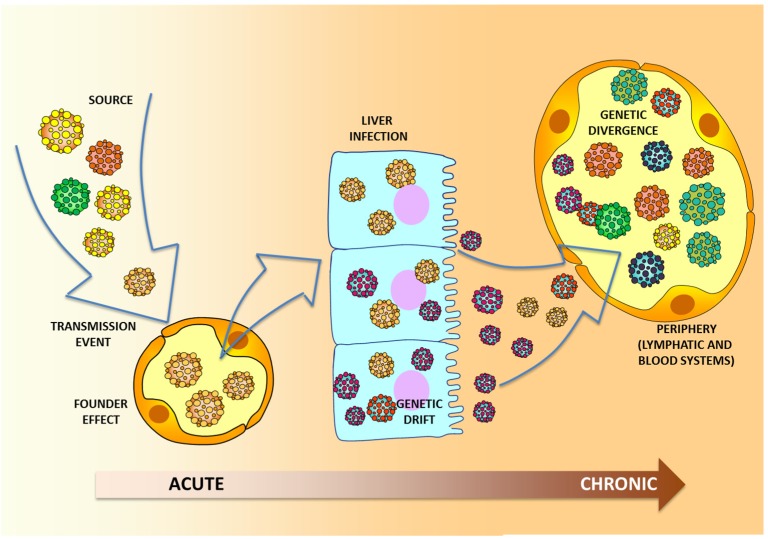
HCV intrahost molecular evolution. The main forces driving the molecular evolution of HCV are depicted. The generation of viral diversity during the course of natural infection is illustrated. Upon infection, the virus migrates via blood vessels to the liver where replication takes place. Viral diversity is increased throughout the infection process and reflected in the periphery over time.

The molecular mechanisms that shaped the evolution of HCV are quite diverse. Upon transmission, HCV undergoes a series of events that affect the architecture of the viral population (Figure 4). The “steps” taken by HCV throughout the infection process are highly complex and depend on fluctuations in the complexity and size of the intrahost population. HCV transmission events are characterized by genetic bottlenecks that inflict a strong selective pressure during acute infection [56,97,98,99]. Indeed, the HCV genetic bottlenecks are so intense following transmission that very few variants are able to establish infection in the new host [100,101]. Subsequently, genetic drift commonly takes place as the main force driving the molecular evolution of HCV during the early stages of the acute phase as a result of the strong founder effect [102,103,104]. During the later stages of HCV infection the extent of genetic drift is significantly reduced [102,103,104,105], allowing a large viral population size to come to prominence in chronic cases. To a lesser degree, genetic recombination also affects overall HCV genetic heterogeneity. HCV genetic recombination is rare [106], mainly due to the exclusion of superinfections [107,108,109]. However, despite limited HCV recombination, naturally occurring inter-genotype, intra-genotype, and intra-strain HCV recombinants have been reported [110,111,112,113]. Thus, HCV recombination might have important implications for clinical and epidemiological studies [114]. Staging of HCV infections has also been proposed [115]. This hypothesis suggests that during chronic infection, HCV attempts to reach a settlement stage characterized by a viral population undergoing strong negative selection. This “journey” involves complex processes including temporal variations attained by incremental changes between communities, random mutations and fluctuations in the frequency of coexisting viral subpopulations; in conjunction these factors constantly reshape the architecture of the viral population [89,95,115,116]. This staging is likely to affect HCV transmission because different viral variants that most likely possess different degrees of transmissibility are available at different time points during the course of the infection [21]. Despite the complexity of the means used by HCV to evolve in an adverse microenvironment, genetic relatedness can be successfully established in many instances if transmission has occurred relatively recently. In addition to time of infection, establishment of relatedness depends on the degree of divergence and other external factors such as a history of antiviral therapy, which can significantly alter the natural course of HCV evolution.

**Figure 5:**
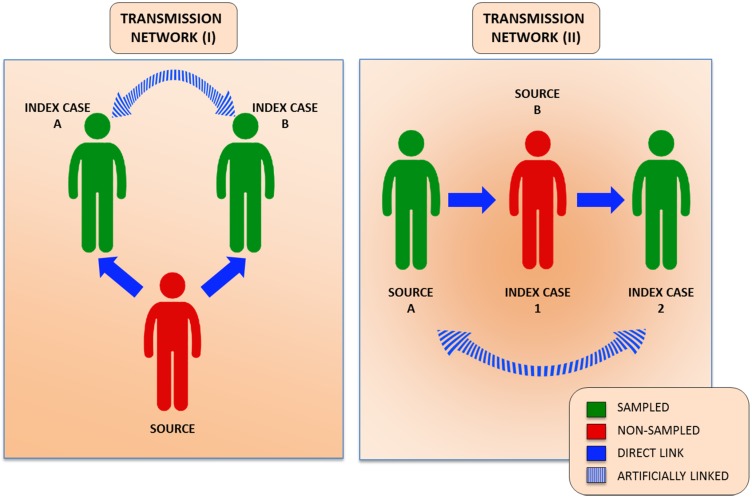
HCV transmission networks. Two transmission networks are depicted. The limitations of HCV molecular studies are depicted when not all individuals belonging to the transmission network are included. Artificially linked cases can be observed when not all members of the transmission network are sampled.

HCV transmission networks are difficult to be recognized for numerous reasons [117,118]. The long incubation periods and the characteristic asymptomatic nature of acute HCV infections make identification of cases a rather challenging task [117]. HCV transmissions are difficult to link to their corresponding source of infection because intrahost viral populations are often genetically related but seldom identical [95,119]. Importantly, branching in phylogenetic analyses, derived from genetic relatedness studies, do not always correspond to transmission events, particularly in those cases where not all individuals belonging to the transmission network are sampled (Figure 5). Thus, local epidemic sequences can cluster together in the absence of direct transmission [119,120].

HCV evolution is also affected by preexisting liver conditions, infection with other viruses and also pregnancy as a direct consequence of the alterations of the immune response in the mother [17]. During pregnancy, increased viral loads and a reduction in CD8+ T cell cytotoxicity are commonly observed, favoring the emergence of more fit strains [121] as a result of the impairment of the antiviral response in the mother. Therefore, children infected perinatally might potentially bear infections with the more fit viruses, thereby facilitating persistence [122].

## 4. Human Genomics in HCV Advanced Molecular Surveillance

The era of human genetics is expected to play an important role in the clinical management of infectious diseases [123]. HCV infection is not the exception, and several studies have shown that host genetic makeup affects the outcome of infection and therapy [21].

HCV is spontaneously cleared by the host in up to 15% of acutely infected individuals [124,125,126]. It has been reported that an early functional inactivation (peripheral tolerance) of the HCV-specific CD4+ T cell response may play a major role in establishing viral persistence [127,128,129,130], and such dysfunctional T cells can be functionally reactivated [131,132]. Several studies have suggested that differences in host immune responses determine viral clearance. Indeed, the intensity of the immune response can be dictated, at least in part, by host genetic factors [133]. However, the role of host factors in HCV clearance is not well understood. Different studies have shown correlations between HCV-related disease and the genetic background of the host [75,134]. For example, certain HLA alleles have been associated with specific outcomes of HCV infection [135,136]. As a result of the completion of the Human Genome Project and the development of novel methodologies capable of rapidly assessing the host genetic background, a number of disease-associated genes have recently been reported. Consequently, mutations in these genes can be screened and used to assist in the clinical decision making process [137]. This information can also be incorporated into heightened surveillance for a number of infectious diseases, facilitating the incorporation of pharmacogenetics into global surveillance [138].

Recently, several single nucleotide polymorphisms (SNP) in immune-related genes have been associated with HCV disease outcomes. Large-scale genetic analyses have suggested the participation of different SNPs in the spontaneous clearance of HCV, including TNFSF18 (tumor necrosis factor superfamily, member 18), TANK (TRAF family member associated NF-κB activator), HAVCR1 (Hepatitis A virus cellular receptor 1) and IL-18BP (IL-18 binding protein) [139]. Additionally, SNPs occurring in the gene encoding tumor necrosis factor alpha (TNF-α) have also been proposed to participate in HCV clearance, particularly in subjects of African origin [140,141]. TNF-α is an important cytokine produced primarily by macrophages that participates in the induction of apoptosis, gene regulation and cellular proliferation [142]. Interestingly, high levels of TNF- α have been detected in the serum and liver of chronically infected patients [143]. SNPs in transforming growth factor (TGF)-β1 and IFN-γ have also been suggested to participate in HCV clearance [144,145]. TGF-β1 is a suppressor of natural killer (NK) cells that inhibits the production of IFN-γ and IL-12, thereby blocking the proliferation and cytotoxicity of NK cells [146]. Moreover, dysregulation of TGF- β1 has been shown to be involved in the progression of liver cirrhosis and HCC [147]. IFN-γ is a multifunctional cytokine produced by effector T and NK cells that participates in the development of T helper 1 (Th1) cells and is critical for host defense against a variety of intracellular pathogens, including HCV. IFN-γ inhibits HCV replication *in vitro*, and intrahepatic levels are associated with viral clearance in animal models [148,149]. SNPs in immune-related genes encoding for interleukin-10 (IL-10) have been reported to play role in HCV-related infection [141]; however, the role of IL-10 in HCV infection is debatable. IL-10 inhibits IFN-γ production, resulting in an imbalance in the T helper response that in turn leads to viral persistence [150], while low levels of IL-10 seem to be associated with resolution of HCV infection [151]. Conversely, IL-10 has been proposed to be antifibrogenetic in chronic liver injury [152].

SNPs in the osteopontin (OPN) gene (−1748 and −443) have been associated with chronic HCV infection [153]. Response rates were higher in patients with the G/G or G/A alleles at nt −1748 in comparison to patients bearing the A/A alleles. Likewise, the response rate was higher in patients with the T/T alleles at nt −443 than in those with C/C or C/T alleles [153]. OPN is a highly phosphorylated sialoprotein and an important component of the extracellular matrix [154], which is secreted by lymphocytes, leucocytes, and macrophages. OPN interacts with cellular adhesion molecules and plays a role in different aspects of the cellular immune response, but the exact role of OPN in HVC infection is still unclear.

Recent studies have demonstrated an association between a SNP in the exon 7 splice acceptor site of the oligoadenylate synthetase 1 (OAS1) gene and SVR in HCV patients. Patients exhibiting an AA genotype showed poor SVR rates and progressed to more severe disease [155]. OAS1 is an important protein with antiviral activity [156] that is activated by double-stranded RNA. Upon activation, OAS1 inhibits viral RNA and protein synthesis. Thus, nucleotide changes affecting its function are expected to further impair virus infection control.

Importantly, a SNP in the mannan-binding lectin (MBL) gene (also known as the mannose binding protein) has been related to HCV infection. HCV patients tend to exhibit YA/YO in comparison to controls and reduced levels of MLB in plasma [157]. Additionally, the frequency of these genotypes is reduced in patients with advanced fibrosis compared to patients with moderate fibrosis. MLB is a pattern recognition receptor (PRR) that has an important function in the innate immune response [158]. Nevertheless, its role in HCV control is not well known.

Another host factor associated with HCV infection is the occurrence of SNPs in the promoter region of the IL-28B gene (also known as interferon-λ 3). The type III IFN family includes IFN-λ 1, 2, 3 and 4 (IL-29, -28A, -28B, IFN-λ 4, respectively) [159,160]. Based on their molecular structures type III IFNs belong to the interleukin-10 (IL-10) superfamily, but functionally they are closely related to type I IFNs (IFNα, IFNβ), which play a major role in antiviral immunity [161]. IFN-λs are produced by dendritic cells, neuronal cells, alveolar epithelial cells, and hepatocytes [162], in response to viral infections through its activation via Toll-like receptors (TLRs) [163]. IFN-λs inhibit viral replication and modulates immune-related functions, such as the maturation and differentiation of immune cells [164,165,166]. The SNPs in IL-28B have been recognized as strong predictors for both spontaneous and antiviral-induced clearance of HCV. Several studies have shown that patients infected with HCV genotype 1 bearing the C/C, A/A and T/T alleles in rs12979860, rs12980275, and rs8099917, respectively, are more likely to achieve SVR [75,76,77]. Recently, the discovery of a new gene within the type III IFN family (IFN-λ4) situated upstream of IFNL3, and its association with a poor response to IFN-α therapy and impaired HCV clearance suggests a more complex role for the interferon response in HCV infection [160]. However, the arrival of second and third generation treatment regimens based on combinations of different DAA with and without IFN has down-played the relevance of these SNPs in the prediction of SVR.

## 5. Assessment of the HCV Intrahost Genetic Variability

DNA sequencing is essential for the study of transmission, phylogenetics, and outbreaks [167]. Upon transmission, a small subset of the HCV population from the source is transferred to a new host (recipient) and then subjected to immune pressures based on the new host’s genetic background. Therefore, the viral populations in the two individuals are related but genetically distinct [88,89,95,96]. Additionally, the characterization of viral variants is also useful for the study of virus tropism in host compartments [89]. The selective forces driving the molecular evolution of HCV are complex in nature and difficult to assess. Most molecular approaches, such as population sequencing, required for molecular characterization of HCV intrahost populations, are arduous, lengthy and costly [168]. However, new technologies based on NGS platforms allow a finer resolution and are more affordable [59,67,168,169], facilitating the molecular characterization of HCV intrahost viral populations [64,168,170].

The analysis of the composition of HCV intrahost populations requires nucleotide sequencing of as many variants as possible in the patient. The assessment of the complexity of the HCV intrahost population is challenging due to the difficulty in detecting low-frequency or minor variants. Establishment of genetic relatedness is affected by the variability of the genomic region analyzed, the time since transmission and the length of the subgenomic region. Moreover, sequence errors introduced by the reverse transcriptase and DNA polymerase during amplification of the template can occur. HCV population complexity can also be skewed by template re-sampling during PCR in low titer clinical samples, resulting in apparently false reduced complexity. Additionally, major variants may mask minor variants due to their respective frequencies during re-sampling. Different approaches have been developed to characterize the HCV intrahost population with different inherent limitations. Alternatives approaches developed for HCV intrahost characterization are based on the identification of nucleotide sequences or assessment of the diversity and complexity without the need to sequence the viral genome [171,172]. Complexity, cost and reliability are some of the factors that play a role in the choice of methods. Historically, some methods were not powerful enough or presented too many shortcomings to overcome. Consequently, these methods have been disused and replaced by more powerful methods capable of analyzing the composition of the HCV intrahost population to an unprecedented level.

Non-sequencing-based methods are disadvantageous because the structure of the viral population is not obtained and the performance relies on the DNA properties of the variants being analyzed. In contrast, sequence-based strategies provide a high-resolution snapshot of the circulating viral variants. Several challenges are faced during the implementation of these techniques, including the stochastic nature of PCR-based methodologies that can significantly distort the composition of the HCV intrahost population.

### 5.1. Genomic Regions

Tracking of HCV infection depends on sequence information originated from different subgenomic regions. The 5'-UTR region has been widely used for detection owing to its degree of conservation across genotypes, while the NS5B region is the most common target for HCV genotyping [173,174]. However, these two regions are well conserved and, therefore, do not contain sufficient sequence information to establish genetic relatedness between clinical isolates. Instead, genetic relatedness studies primarily rely on information obtained from HVR1 [16,89,93,95]. Rapid divergence in this region represents a challenge for molecular epidemiological studies and can result in the loss of genetic links between related isolates [16]. Sequencing of multiple and longer subgenomic regions has been proposed as an alternative to overcome the limitations imposed by the rapid molecular evolution of HCV [16]. NS5A has been used to establish relatedness among HCV cases [115]; the use of this gene can restore links between isolates owing to its lower nucleotide substitution rate. Despite the usefulness of different subgenomic regions for the characterization of clinical isolates, whole genome sequencing should be the ultimate goal for HCV molecular epidemiology.

### 5.2. Single-Stranded Conformation Polymorphism (SSCP)

Prior to the implementation of more sophisticated molecular methods for the characterization of individual variants, SSCP was a relatively popular alternative [175,176,177]. This approach is based on the denaturation of single-stranded amplicons that are resolved in non-denaturing conditions to differentiate molecules based on their secondary structure [178]. The popularity of this method was originally based on the assumption that different variants may adopt different secondary structures; however, several studies showed the implicit limitations of SSCP for the identification of closely genetically related variants [179]. Sensitivity is another major limitation of SSCP, because only variants present in >3% of the viral population can successfully be identified [180], and therefore, minor variants are unlikely to be detected. Additionally, the length of the regions studied imposes important limitations, because long fragments may not resolve as clearly as shorter fragments [181]. Other factors affecting the performance of SSCP include DNA concentration and GC content. Despite being an inexpensive and relatively straightforward approach, SSCP lacks the required resolution to accurately assess the composition of the HCV intrahost population. Thus, it is not surprising that its use has diminished with the arrival of more powerful technologies better suited for such demanding task.

### 5.3. Heteroduplex Gel Shift Assays

In many ways, heteroduplex gel shift assays resemble SSCP [182,183], and therefore, the benefits and disadvantages between the two methods are rather similar. Overall, the entire intrahost population can be sampled; however, no specific information about specific substitutions in the population can be obtained. This methodology is based on the amplification and hybridization of the viral RNA to a labeled probe. Next, the heteroduplex products are resolved by electrophoresis. Thus, different variants are expected to exhibit different migration patterns due to mismatches with the probe, resulting in differential band profiles depending on the composition of the intrahost population. A slight variation in the conventional heteroduplex assay relies on the cloning of different viral species into plasmids instead. Genetic differences can be calculated using a heuristic approach known as the heteroduplex mobility ratio. However, the accuracy provided by this method is limited and usually requires variants to differ by ~1.5% [182].

### 5.4. Population Sequencing

#### Bacterial Cloning

Sequencing individual variants is a cumbersome and costly task that provides important information about the composition of the intrahost population. The basis of this approach includes amplification of the region of interest, followed by cloning of the amplicons into a plasmid vector and transformation of bacteria. Then, the transformed bacteria are plated. The bacteria are assumed to carry only one plasmid, and the presence of only one viral variant sequence is confirmed by colony PCR and sequencing. Thus, DNA from an individual bacterial colony represents the sequence of a single variant. Unfortunately, sampling is a major issue for this particular approach because even in large studies that analyze hundreds of clones, only a small fraction of the viral population and genome is sampled [173]. Moreover, the exact number of clones necessary to achieve a given level of confidence depends on the frequency of each incorrect nucleotide at any given nucleotide position in the sequence [184]. It has been suggested that between 20 and 100 clones per sample are required for frequency and entropy analyses. Sequencing 99 clones has been proposed to identify 95% of all variants present at a frequency of at least 3% in the population [185]. However, others have suggested that sequencing 20 clones is sufficient to cover 95% of the major variants (frequency >10% in the population). Regardless of the estimate, under these circumstances, most minor variants go undetected, significantly hampering our capacity to untangle the mechanisms involved in HCV evolution. Recent advances in single genome sequencing have improved our understanding of HCV transmission [101]. In summary, regardless of the number of clones analyzed, the depth with which population sequencing can assess the complexity of the HCV intrahost population is extremely limited.

### 5.5. End-Point Limiting-Dilution PCR (EPLD-PCR)

EPLD-PCR is a viable alternative to single-molecule PCR amplification and has largely been used to identify individual viral variants of different viruses including human immunodeficiency virus (HIV), hepatitis B virus (HBV) and HCV [186,187,188]. EPLD-PCR relies on the isolation of individual coexisting sequence variants in clinical samples using a limiting-dilution approach followed by sequencing. Overall, EPLD-PCR is less prone to distortion compared to bacterial cloning-based methods because the amplification is based on single DNA molecules. Moreover, real-time-based EPLD-PCR has been shown to be superior to conventional population sequencing methods for the detection of HCV viral variants. However, implementation of EPLD-PCR is cumbersome and time consuming, resulting in a rather expensive methodology.

### 5.6. Mass Spectrometry (MS)

MS was developed as an alternative to the numerous DNA sequencing technologies [171]. Originally, the accuracy, sensitivity and high-throughput methodology of MS set the basis for the implementation of molecular methods aiming to characterize single nucleotide changes in DNA molecules [189]. MS analysis relies on the measurement of the molecular masses of different analytes, which should be ionized and separated before detection. Unfortunately, ionization may lead to partial degradation of the analytes, thereby affecting the resolution of this method. To minimize the effect of ionization on the analytes, methods such as matrix-assisted laser desorption/ionization and electrospray ionization have been developed [190,191]. MS has been used for resequencing, microbial typing, and single nucleotide polymorphism discovery [172,192,193].

Analysis of the HCV intrahost population by MS has been reported [172,192,194]. MS represents a high-performance methodology that analyzes base-specific cleaved RNA originating from PCR fragments [171,172,195]. This is a cost-effective and reproducible approach capable of high throughput. The resolution of this method easily accomplishes the identification of nucleotide polymorphisms comprising >10% of the amplicon population. The data originating from this type of analysis represents a composite pattern of the variants present in the sample [36]. Importantly, the identification of novel single-nucleotide variants depends on the proper representation of such variants in the reference database required for base calling [196]; therefore, a constant update of the reference sequences is required. Despite their implicit advantages, MS-based approaches have not been largely used for assessment of HCV intrahost populations based on the fact that *de novo* sequencing is challenging and also because of the arrival of more powerful and inexpensive methods [173]. 

### 5.7. Next Generation Sequencing (NGS)

The advent of (NGS) has revolutionized the diagnostics of infectious diseases [197]. The development of massively parallel ultra-deep pyrosequencing allows for a high-resolution snapshot of the intrahost viral population. Additionally, newer approaches have overcome the issue associated with short reads, resulting in longer sequences that facilitate the reconstruction of the viral population [93,173,198,199,200].

Ultra-deep sequencing is based on a limiting dilution approach and allows rapid sequencing of a large number of variants by eliminating the need to separate molecules and clone into bacterial vectors. Ultra-deep sequencing has been used to identify minor variants in different settings [59,201].

Different platforms are available, including 454 Life Science (Roche), Illumina, Ion Torrent and Pacific Biosciences. These platforms were initially developed as an alternative to the prohibitively expensive Sanger method. Comprehensive characterization of viral populations is easily accomplished with NGS. The advantages of NGS technologies in virology are numerous. Metagenomics is a growing field in virology that has allowed the characterization of viral populations from different types of samples [202,203,204], especially the detection and characterization of viruses associated with disease outcomes [205]. However, overcoming different challenges is required for the implementation of NGS approaches in the study of viral diseases.

NGS amplicon sequencing allows sufficient coverage in order to detect variants occurring with a frequency of <0.1%. The low abundance of nucleic acids is a major problem for the molecular characterization of viruses from clinical samples. The low ratio of viral RNA/host RNA commonly observed in clinical samples is one the main obstacles to using NGS methods. Pre-amplification and probe capture are some of the alternatives available to overcome the relatively low frequency of viral RNA commonly present in clinical specimens. Additionally, viral enrichment and concentration have been used as alternative methods to improve sequencing quality and depth. Nevertheless, whole genome (WG) viral sequencing remains challenging. The analytical sensitivity of WG using NGS approaches is not as easy to evaluate and largely depends on the depth of sequencing [206,207]. However, increasing the depth of sequencing for an optimized sample preparation can decrease the level of detection. Moreover, the analytical sensitivity depends on the length of the genome. Longer lengths translate into a higher number of potentially available reads, as seen for some studies of viruses. This should also be the case for bacterial and fungal genomes, which could be viewed as an advantage for the detection of such microbes because their concentrations in blood can be very low even in samples from infected patients. Several studies have evaluated the diagnostic sensitivity of this technique. Analysis of the human virome in febrile and afebrile children allowed the identification of a wide range of viruses that correlated with the outcome of disease, as well as virus subtyping [208]. Another advantage of the technique is its capacity to identify co-infections, which is important for the development of adaptive therapeutics [197].

Thus, NGS will likely become a routine test for diagnostics of infectious diseases [209]. For this initiative to succeed, improvements in sample preparation, availability of sequencers in central laboratories and validated pipelines for read sorting and taxonomic assignation must be attained. The study of viral genetics in the era of next generation sequencing promises to help to unveil the most intimate details of the virus-host interface [168].

## 6. Bioinformatics, Phylogenetics and Data Mining

### 6.1. Bioinformatics

A variety of computational challenges are commonly encountered in the field of virology owing to the high diversity of viruses, their compact genome organization and their rapid rate of evolution [210]. For HCV, the increasing number of viral sequences available in public and private databases has promoted the development of novel ways to analyze considerably large data sets [211,212]. Relatively recent technological innovations have ignited an explosion in virus genome sequencing that is likely to help us understand the intimate details of HCV biology and its impact on public health. Nonetheless, any benefits derived from the generation of massive sequence data are hindered by the implementation of resources capable of identifying the sequences, as well as assembling, annotating, curating, maintaining and storing extremely large databases [117,211,212]. Importantly, virology has recently drawn the interest of the bioinformatics community, which in turn has led to the development of a variety of tools. This rapid expansion of the HCV sequence universe has forced a recalibration of the data model to better provide extant sequence representation and enhanced reference sequence products to serve the needs of the various viral communities [211,212,213]. However, despite the growth in viral bioinformatics, a number of questions remain unanswered, including but not restricted to identification, genome annotation, phylogeny, evolution, and genetic diversity.

Viral evolution has many implications for clinical virology. The emergence of HCV resistance mutations is one of the most important challenges for successful antiviral therapy [214]. The molecular mechanisms selecting resistance mutations are complex; therefore, new bioinformatics approaches to characterize HCV evolution both at an intra- and interhost level are required. The integration of bioinformatics methods might lead to predictions of viral evolution in HCV chronic patients based on sequences derived from their intrahost viral populations. Hence, the ultimate goal in HCV genetics is the prediction of the course of HCV evolution that in turn could lead to the customized management of the patient and hepatitis C treatment.

### 6.2. Phylogenetics

Viral phylogenetics have also benefited from the exponential growth of bioinformatics. Phylogenetics is an important area in virology, particularly in HCV molecular epidemiology [21]. However, several characteristics of HCV are challenging for phylogenetics. Significant differences in evolution rates (high over the short term and much lower over the long term), gene transfer, evolutionary virus-host relationship, and the lack of physical “fossil records” of viruses (ancient viruses) remain difficult obstacles to overcome. Additionally, phylogenetic trees derived from HCV sequence analyses cannot faithfully represent complex evolutionary relationships relevant to HCV such as horizontal gene transfer, recombination or evolutionary virus-host relationships. Novel phylogenetic approaches have been developed in recent years to better represent such relationships [215,216]. However, the field of phylogenetics warrants further research to address several aspects related to the reconstruction of HCV phylogeny.

Establishing the time of infection is one of the most important and challenging tasks in any epidemiological investigation. However, infection metrics among cases with multiple risk factors and possible exposures are difficult to pinpoint. Therefore, determination of time of infection using molecular data represents an attractive line of research. Estimating the time of infection based purely on genetic data has been reported [92,217,218]. However, molecular clock estimates vary significantly, and their reliability depends on a number of factors including sampling, temporal and anatomical distribution of sampling, genome region sequenced, super- or re-infection, and the evolutionary models and algorithms used [119]. As a consequence, the implementation of such approaches for genetic relatedness studies should be undertaken cautiously [119].

### 6.3. Databases and Data Mining

A database is a collection of data that is organized so that its contents can easily be accessed, managed, and updated. Many databases for infectious diseases compile data obtained from clinical and/or public health laboratories, aiding the monitoring of infectious disease trends and surveillance [219,220]. However, even with our growing ability to acquire sequence data, its potential to impact disease surveillance is only fully accessed when data are translated into public health actions.

Initiatives such as the Viral Bioinformatics Resource Center (VBRC) aim to close the gaps between massive viral sequencing and bioinformatics are a welcome addition to the set of on-line bioinformatics tools available to the scientific community. These tools provide comprehensive web-based genomics resources that are useful for basic and applied virology research. The VBRC consists of relational databases and web applications for data storage, annotation, analysis, and information exchange. Curation of the data results in the ability to search for gene functions relating to biological genotypes and phenotypes with an emphasis on pathogenesis and provides a variety of analytical and visualization tools [213].

The HCV Database Project was initially funded by the Division of Microbiology and Infectious Diseases of the National Institute of Allergies and Infectious Diseases (NIAID). The HCV database is a resource for the scientific community working on HCV genetics, evolution, variability, and vaccine and drug design [211,212]. The HCV sequences deposited in GenBank composed the backbone of the database, including information such as country, sampling year, isolate names, genotype and subtype, and host species in addition to relevant annotation information associated with the corresponding publications. Annotation fields in the database include genotype, subtype, start and stop coordinates relative to the reference strain HCV-H, sampling country, sampling city, sampling date and sampling tissue. Patient information is also documented in the database, including health status, age, gender, ALT level, treatment and result, co-infection with HIV and hepatitis B, infection date, infection country, infection city, infection route, infection outcome, HLA type, and epidemiological relationship with other patients. The HCV database contains HCV-associated, hand-annotated genetic data and provides access to the central database via web-accessible search interfaces in addition to a number of analysis tools. The Los Alamos HCV database emerged as a result of the success of the HIV Database Group and uses a similar approach [212]. The infrastructure developed for the dynamic alignment of a large numbers of sequences in the HIV database was quickly implemented for the HCV database [221]. Tools designed for the manipulation of viral sequences (gene extraction, coordinates to reference strains, sequence alignment, and nucleotide or protein motifs scanning) were easily adapted for the HCV database. The information is accessed via an interface allowing for advanced searches with sorting and graphical overview capabilities. Importantly, sequence data can be retrieved as a DNA alignment. Pre-made, manually optimized alignments are also provided in a variety of flavors. Synonymous/non-synonymous substitution analysis for all genes and proteins is available. The Geography tool can plot genotype frequencies based on their geographical origin. Other functions include glycosylation site identification, *Modeltest*, *Treemaker*, *BLAST*, *PCOORD*, *Gene Cutter*, *Consensus*, *PeptGen*, *Motiscan*, *Primalign*, *Epilign*, *Seq-convert*, *OmniRead*, *SeqPublish* and *Sequence locator* [211,212]. Unfortunately, this valuable resource is no longer funded by the NIH; therefore, the herculean effort to properly maintain it has been significantly delayed by the lack of financial support.

The Virus Pathogen Database and Analysis Resource (ViPR) supports virology researchers studying select agents and other significant public health pathogens belonging to 14 virus families including HCV [222]. Cross-referencing data and integrated computational tools into the online ViPR resource allow complex analyses. ViPR captures data from external and internal sources and makes them accessible through custom searches. ViPR is primarily focused on viruses of human interest; however, families isolated from other host species are also available for comparison. ViPR features a suite of data analysis and visualization tools to perform custom correlative analyses. ViPR uses the NCBI RefSeq strains to extend the manually optimized annotations to the rest of the taxon. Thus, the ViPR resource provides the scientific community with friendly tools capable of performing complex analytical workflows [222].

**Figure 6:**
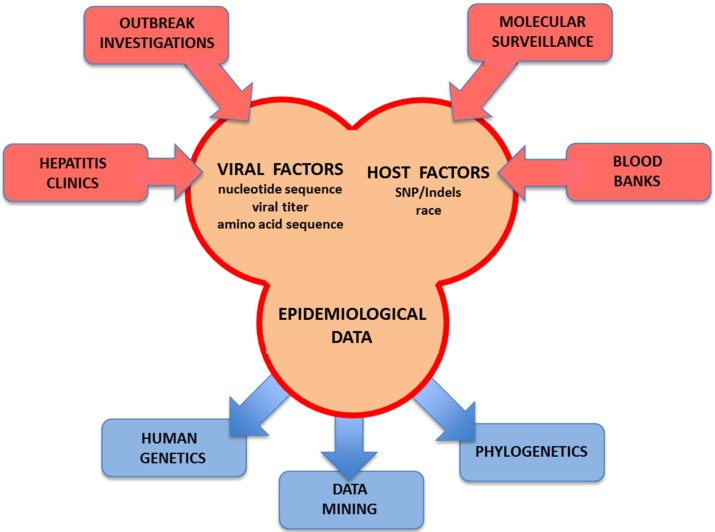
Advanced HCV molecular surveillance. The main components required for advanced molecular surveillance of HCV are listed. Global databases containing comprehensive information concerning transmission events are used as a source of information to draw conclusions about viral spread. Advanced molecular tools and data mining analyses are required to accurately identify the nuances driving the transmission of HCV.

Importantly, the requirement for the establishment of a database-type of molecular surveillance network that is feasible for international surveillance networks is the standardization of the input data. Several initiatives involving international partnerships and pilot studies have been initiated for an array of pathogens including HCV [219,223,224]. Data sharing is critical for an interdisciplinary approach in order to tackle a problem [223]. The introduction of NGS in routine diagnostics is likely to further boost HCV integrative surveillance. By applying analytical tools to genomic data for HCV, public health scientists can track specific mutations that confer the ability to resist drugs or link them to transmission networks. However, the information provided by raw genomic sequences of pathogens must be integrated with knowledge about the host biology as well as societal and environmental factors in order to understand the etiology of epidemics and to anticipate their trajectories [223]. Therefore, the development of a diagnostic pipeline critically relies on database exhaustiveness that can match the rapid growth of databases prompted by NGS. A typical blast analysis of millions of sequences after *de novo* assembly into larger *contigs* against the whole NCBI database using relaxed criteria is time- and resource-consuming for diagnostics. In contrast, stringent mapping of non-assembled reads in a comprehensive database in conjunction with long sequence reads will likely improve the overall process. Thus, an HCV integrative molecular surveillance tool should ideally be web-based and include both viral and host factors as well as epidemiological data (Figure 6) [220]. In addition to international public and private sequence databases, the ideal HCV integrative database should be enriched with data obtained from ongoing molecular surveillance projects and outbreak investigations, supplemented if at all possible with sequence information from cases identified in hepatitis clinics and blood banks [117]. As a result, comprehensive analyses including human genetics, phylogenetics and data mining could be easily attained locally by researchers in the field with minimal effort at an affordable cost for local state public health laboratories (Figure 6).

Data mining, or “Knowledge Discovery in Databases”, is an interdisciplinary subfield of computer science referring to the computational process of discovering patterns in large datasets via artificial intelligence, machine learning, statistics, and database systems [225]. The ultimate goal of data mining is the analysis of tremendous volumes of data to discover hidden patterns and relationships in highly complex datasets, rendering valuable information. This is attained by a series of data pre-processing, model and inference considerations, interestingness metrics, complexity considerations, post-processing of discovered structures, and visualization processes [225]. The application of machine learning methods and advanced statistical modeling to laboratory data and the subsequent discovery of patterns is of relevance for the identification of factors associated with disease outcomes [226]. For HCV infection, decision-tree analysis (a core component of data mining analysis) was used to build predictive models for therapeutic outcomes to antiviral therapy in chronic HCV patients and the severity of disease [227,228,229,230,231]. Decision-tree analysis relies on a tree-shaped structure representing decision “calls” with classifying power, where each internal node denotes an attribute, each branch represents an outcome of the test, and each terminal node a class [227]. Decision-tree analysis facilitates the classification of patients into subgroups that can identify the possibility of an outcome of HCV therapy and, thereby, improve diagnostics [232,233,234,235].

Other approaches, such as linear progression and Bayesian network analyses, have also been shown to produce robust predictions for the progression to fibrosis [229]. Interestingly, HCV epistasis is strongly associated with host factors [230], suggesting that intrahost viral evolution is convergent and that important traits can be modeled, resulting in predictions with clinical relevance.

## 7. Concluding Remarks

HCV molecular evolution in many ways affects virus spread and disease transmission. The sophistication achieved by HCV in shaping its molecular evolutionary patterns is intricate and has significantly hindered our ability to fully understand the mechanisms exploited by the virus to ensure transmission. The remarkable HCV mutation rate represents a challenging task for molecular epidemiology. In this new era of advanced sequencing technologies, the implementation of enhanced molecular surveillance is of the utmost importance in order to accurately monitor the circulation of viral strains. Comprehensive molecular studies are required to uncover the key participating elements responsible for virulence. Supplementing molecular data with epidemiological information and host factors significantly improves the accuracy of HCV molecular surveillance. Therefore, the fact that comprehensive epidemiological investigations must be carried out cannot be understated.

Improved assessment of HCV intrahost genetic variability should clarify the pathway towards advanced integrative molecular surveillance and ultimately help to unveil the mechanisms driving viral transmission. For advanced molecular surveillance to be truly effective, implementation of preventive and control measures along with therapeutic interventions should be performed. The past 25 years since the discovery of HCV have been quite a journey, and the future of HCV control looks more promising than ever before.
